# Performance Enhancement Method for Angular Rate Measurement Based on Redundant MEMS IMUs

**DOI:** 10.3390/mi10080514

**Published:** 2019-08-01

**Authors:** Li Xing, Xiaowei Tu, Weixing Qian, Zhi Chen, Qinghua Yang

**Affiliations:** 1 School of Mechatronic Engineering and Automation, Shanghai University, Shanghai 20444, China; 2 College of Electric and Automation Engineering, Nanjing Normal University, Nanjing 210023, China

**Keywords:** redundant IMUs, MEMS, gyroscope, angular rate measurement, data fusion

## Abstract

Aiming at the low-cost, wide-range, and accurate measurement requirement for Microelectromechanical System (MEMS) Inertial Measurement Unit (IMU) on a multi-rotor Unmanned Aerial Vehicle (UAV), the paper designs a heterogeneous parallel redundancy configuration scheme. In redundant MEMS IMUs, a high-cost and small-range MEMS gyroscope is combined with low-cost and large-range MEMS gyroscopes. Then, an adaptive data fusion method of redundant MEMS gyroscopes is proposed. By the designed experiments based on the simulation data and the sensor measurement data, the proposed method has been proved that it can effectively improve the angular rate measurement performance of the multi-rotor UAV and broaden the angular rate measurement range on the basis of saving the configuration cost and volume of the micro IMU.

## 1. Introduction

Motors and propellers in multi-rotor Unmanned Aerial Vehicles (UAVs) are directly fixed on a frame. The vibration coupling caused by the rapid rotation of propellers will make the flexible frame deform, causing an extremely complicated airborne vibration environment [1,2,3,4]. In order to ensure the stability of the flight control system in the complex airborne vibration environment, especially the stability of the attitude loop control, the multi-rotor UAV puts forward higher requirements on the angular rate measurement range and accuracy of the Microelectromechanical System (MEMS) gyroscope equipped in the navigation control system. A sufficiently large angular rate measurement range is the basic premise for multi-rotor UAV control reliability and anti-jamming capability [5]. The angular rate accuracy and reliability of industrial-grade MEMS gyroscopes [6,7,8] are significantly higher than those of the consumer grade, but its range is generally narrower, which makes it difficult to meet the wide-range measurement requirements of multi-rotor UAV [9]. To improve the measurement accuracy and broaden the measurement range of the MEMS gyroscope in the multi-rotor UAV, a redundant configuration composed of a plurality of MEMS gyroscopes can be employed [10].

Research on the redundancy configuration technology of MEMS IMU includes the design of a redundant configuration scheme [11] and the redundant information fusion method. In the literature [12], three MEMS Inertial Measurement Unit (IMU) redundancy configuration schemes based on an improved array layout are designed to enhance the performance of MEMS IMU. In the first configuration, the MEMS IMU sensitive axes are placed in the opposite direction to reduce the systematic error of the same type of sensor. In the second configuration, MEMS IMUs with different properties are respectively arranged in horizontal and vertical planes, to enhance the performance of the sensor array. In the third configuration, the redundancy system composed of 18 MEMS IMU arrays is designed in [13], and the performance of the array MEMS IMU is analyzed by Allan variance method. The literature [14] designed a configuration of multiple MEMS IMUs combined with GPS in the pedestrian navigation system, and improved the accuracy of the pedestrian navigation system by using three different data fusion methods. In the literature [15,16,17], the algorithms of error calibration, data fusion, and fault diagnosis are proposed respectively in the designed non-orthogonal MEMS IMU redundancy system.

Through the research of the MEMS IMU redundancy configuration scheme and the redundant information fusion method, angular rate measurement performance of MEMS IMU can be effectively improved. However, when these designs are applied in the multi-rotor UAV, there are specific engineering application issues. This is because the MEMS IMU redundancy design in the multi-rotor drone needs to achieve the following two purposes: (1) Redundant configuration cost, size, and power consumption should be minimized under the premise of expanding angular rate range and improving accuracy. (2) Abnormal output of MEMS gyroscopes in MEMS IMU should be detected and isolated to enhance the reliability of angular rate measurement.

Therefore, based on the above two research objectives, this paper proposes a performance enhancement method for angular rate measurement. The presented method offers the following two advantages over other implementations:

(1) The angular rate measurement range is expanded by designing a MEMS IMU redundant array, which is composed of industrial-grade and consumer-grade MEMS IMUs.

(2) On the basis of saving the configuration cost and volume, the angular rate measurement accuracy and reliability is improved through a robust fusion algorithm for redundant MEMS IMUs.

The performance enhancement method of the angular rate measurement improves the multi-rotor UAV control reliability and anti-jamming capability, and the heterogeneous parallel redundancy configuration of MEMS IMU can also be applied on robotics and self-driving vehicles.

## 2. Angular Rate Measurement Performance Enhancement Method

### 2.1. Design of Parallel Redundancy Configuration Based on Heterogeneous MEMS IMU

Since the multi-rotor UAV has requirements on the volume, power consumption, and cost of the residual MEMS IMU, according to the references in the Introduction, the configuration options available in the redundancy MEMS IMU are: (1) orthogonal configuration using the same type of low-precision sensors; (2) orthogonal configuration using the different type of low-precision sensors; (3) orthogonal configuration using a mix of high- and low-precision sensors. In these types of optional configurations, since the MEMS IMUs configured in (1) are of the same type, their resonant frequency points are close, and the failure modes are similar. When applied in a multi-rotor UAV, there is a problem of simultaneous failure in the case of sudden interference or strong vibration. Thus, it is not suitable as a preferred redundant MEMS IMU configuration used on the multi-rotor UAV. In configuration (2), the sensor type is different, and its reliability is higher than (1), but the sensors are all low precision, so that the accuracy improvement after the redundancy configuration is limited. Based on the above analysis, it can be concluded that the redundancy configuration suitable for the application requirements of the multi-rotor UAV is the orthogonal configuration using a mix of high- and low-precision sensors.

**Figure 1:**
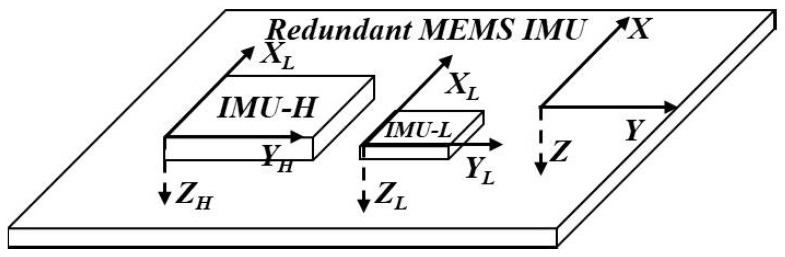
Redundancy configuration diagram with the industrial-grade and consumer-grade Microelectromechanical System Inertial Measurement Unit (MEMS IMU).

In recent years, with the application of MEMS IMU in mobile phones, UAV, and smart electronic devices, many types of consumer-grade low-cost MEMS IMUs have appeared. Although their size and power consumption (e.g., MPU-6000 [18]) are very small and the price is low, their bias stability and bias repeatability are generally poor, so that their measuring accuracy cannot be improved by off-line calibration technology such as industrial high-precision MEMS IMUs. However, the angular measurement range of such MEMS IMUs are generally higher than industrial high-precision ones (e.g., MEMS Gyroscope ADXRS453 [8]). Based on the above analysis, we arranged the industrial-grade and consumer-grade MEMS IMU on the same navigation board shown in Figure 1. In the configuration, the consumer-grade MEMS IMU (MEMS IMU-L) is only a dozen square millimeters in size, but its cost is less than one tenth of that of the industrial-grade MEMS IMU (MEMS IMU-H). Thus, compared with existing redundant configurations, it saves the configuration cost and volume while improving the measurement performance of MEMS IMU.

Based on the designed MEMS IMU redundancy configuration, the gyroscope redundant angle rates fusion method will be researched in Section 2.2 and Section 2.3 to improve the angular rate measurement accuracy and extend the angular rate measurement range.

### 2.2. Redundant Angular Rate Fusion for Online Calibration of MEMS IMU-L Errors

Section 2.2 establishes the redundant angular rate fusion mathematical model, which can calibrate the gyroscope errors of MEMS IMU-L, and then proposes the redundant angular rate fusion method. The fusion model takes the gyroscope bias, scale factor error, and misalignment error of MEMS IMU-L as the estimated states, and the gyroscope output of MEMS IMU-H as the measurement information. Then by adopting the filter algorithm, the gyroscope outputs of different types of MEMS IMU are fused.

#### 2.2.1. State Equation for Fusing the Redundant MEMS Gyroscope Outputs in MEMS IMU

In the state equation, the selected states are shown in (1), where $\left[\omega_{x}^{},\omega_{y}^{},\omega_{z}^{}\right]$ is the fusion angular rate of MEMS IMU-H and MEMS IMU-L, $\left[\varepsilon_{x}^{b},\varepsilon_{y}^{b},\varepsilon_{z}^{b}\right]$ and $\left[K_{gx},K_{gy},K_{gz}\right]$ are bias errors and scale factor errors of MEMS Gyro in MEMS IMU-L [19,20]. In the configuration of Figure 1, there are misalignment errors as MEMS IMU-H could not be completely parallel to MEMS IMU-L, and $\left[\theta_{gx},\theta_{gy},\theta_{gz}\right]$ in Equation (1) denotes the misalignment error angle.

(1) $$x=\left[\omega_{x}^{},\omega_{y}^{},\omega_{z}^{},\varepsilon_{x}^{b},\varepsilon_{y}^{b},\varepsilon_{z}^{b},\theta_{gx},\theta_{gy},\theta_{gz},K_{gx},K_{gy},K_{gz}\right]$$

In Equation (1), the relationship between $\left[\omega_{x}^{},\omega_{y}^{},\omega_{z}^{}\right]$ and the gyroscope output $\left[\omega_{x\_low}^{},\omega_{y\_low}^{},\omega_{z\_low}^{}\right]$ of MEMS IMU-L is given in Equation (2).

(2) $$\left[\begin{array}{c} \omega_{x}^{}\\ \omega_{y}^{}\\ \omega_{z}^{} \end{array}\right]=\left[\begin{array}{ccc} 1-K_{gx} & \theta_{gz} & -\theta_{gy}\\ -\theta_{gz} & 1-K_{gy} & \theta_{gx}\\ \theta_{gy} & -\theta_{gx} & 1-K_{gz} \end{array}\right]\left[\begin{array}{c} \omega_{x\_low}^{}-\varepsilon_{x}^{b}\\ \omega_{y\_low}^{}-\varepsilon_{y}^{b}\\ \omega_{z\_low}^{}-\varepsilon_{z}^{b} \end{array}\right]$$

Based on Equations (1) and (2), the state equation for fusing redundant angular rates is established as shown in Equation (3), where w represents the estimated white noise of fusion angular rate and the subscript k represents the *k*th update process in the filtering period.

(3) $$x_{k}=f\left(x_{k-1}\right)+w$$

The gyroscope bias, scale factor error and misalignment error angle of MEMS IMU-L are modeled as the random constant [20,21].

#### 2.2.2. Measurement Equation for Fusing the Redundant MEMS Gyroscope Outputs

Due to the high accuracy of the MEMS Gyroscope in MEMS IMU-H, its output represents the reference angular rate output of the redundancy MEMS IMU within its range. Therefore, the output of the gyroscope in MEMS IMU-H can be selected as a measurement to directly calibrate the error parameters of the gyroscope in MEMS IMU-L.

The output of MEMS Gyro-H can be expressed as $z=[\omega_{x\_high}^{},\omega_{y\_high}^{},\omega_{z\_high}^{}]$, and the established measurement equation is shown in Equation (4), where v represents measurement noise that is equal to the angular random walk noise of gyroscope in MEMS IMU-H, and H is the angular rate measurement matrix, expressed as Equation (5).

(4) $$z_{k}=Hx_{k}+v$$

(5) $$H=\left[\begin{array}{cccc} 1 & 0 & 0 & 0_{1\times 9}\\ 0 & 1 & 0 & 0_{1\times 9}\\ 0 & 0 & 1 & 0_{1\times 9} \end{array}\right]$$

#### 2.2.3. Angular Rates Fusion Process of Redundant MEMS IMU

In the normal flight state of the multi-rotor UAV, i.e., without special large maneuvering and within the measurement range of the gyroscope in MEMS IMU-H, through the established state equation in Equation (3) and the measurement equation in Equation (4), the fusion angular rate can be estimated and error parameters of the gyroscope in MEMS IMU-L can be calibrated online. The states $\left[\omega_{x}^{},\omega_{y}^{},\omega_{z}^{}\right]$ shown in Equation (2) are coupled to other states, thus Extended Kalman Filter (EKF) is used for state estimation. Error parameters of the gyroscope in MEMS IMU-L are estimated by the output difference of the two types of gyroscopes at the same time, as shown in Equation (6), where $\left[{\tilde{\omega }}_{x}^{},{\tilde{\omega }}_{y}^{},{\tilde{\omega }}_{z}^{}\right]$ is the ideal angular rate and $\left[\omega_{xg}^{},\omega_{yg}^{},\omega_{zg}^{}\right]$ is the measurement white noise. When the UAV has no angular motion, i.e., the ideal triaxial angular rate of the redundant MEMS IMU is zero, only the gyroscope bias is observable. When the UAV only performs angular motion in a single axis, such as ${\tilde{\omega }}_{x}^{B}\neq 0$, the triaxial gyroscope bias, $K_{gx}$, $\theta_{gy}$ and $\theta_{gz}$ can be excited and observed. When the UAV has angular motion on all three axes, all error parameters can be observed.

(6) $$\left\{\begin{array}{c} {\tilde{\omega }}_{x}^{}K_{gx}+\varepsilon_{x}^{b}-\theta_{gz}{\tilde{\omega }}_{y}^{}+\theta_{gy}{\tilde{\omega }}_{z}^{}+\omega_{xg}^{}=\omega_{x\_low}^{}-\omega_{x\_high}^{}\\ {\tilde{\omega }}_{y}^{}K_{gy}+\varepsilon_{y}^{b}+\theta_{gz}{\tilde{\omega }}_{x}^{}-\theta_{gx}{\tilde{\omega }}_{z}^{}+\omega_{yg}^{}=\omega_{y\_low}^{}-\omega_{y\_high}^{}\\ {\tilde{\omega }}_{z}^{}K_{gz}+\varepsilon_{z}^{b}-\theta_{gy}{\tilde{\omega }}_{x}^{}+\theta_{gx}{\tilde{\omega }}_{y}^{}+\omega_{zg}^{}=\omega_{z\_low}^{}-\omega_{z\_high}^{} \end{array}\right.$$

From the above analysis, it can be further concluded that when the redundant MEMS IMU has no angular motion, the non-observed gyro error parameters in MEMS IMU-L would be estimated to diverge. If these parameters were not truncated, they would affect the accuracy of the other measurable error parameters as well as the fusion angular rate.

**Figure 2:**
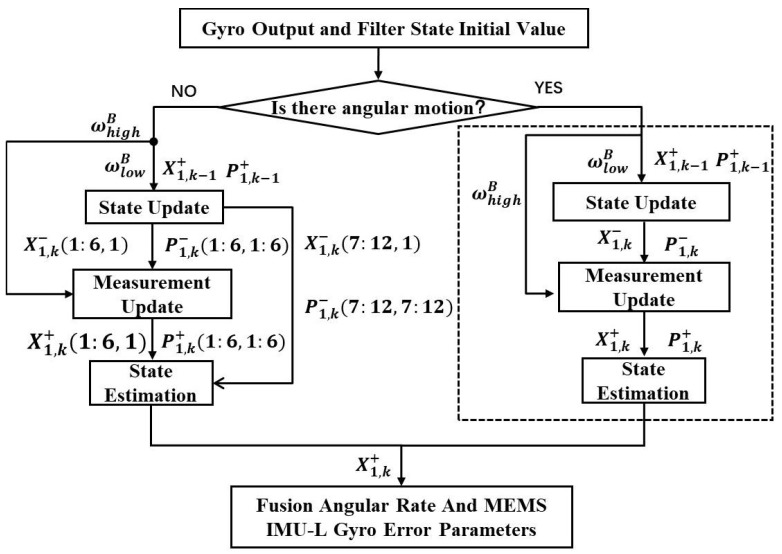
Angular rate fusion process of redundant MEMS IMU.

Therefore, it is necessary at first to determine the angular motion state by the MEMS Gyroscope output before the angular rate fusion. Then, according to different angular motion states, different filtering processes will be used to calibrate the error parameters in the MEMS Gyro-L and fuse the angular rate. Angular rate fusion process of redundant MEMS IMU is shown in Figure 2.

The time update process shown in Figure 2 mainly performs a one-step prediction update of the state and the state covariance, and the update process is as shown in Equation (7). ${\hat{x}}_{k}^{-}$ is the one-step prediction of the state, and ${\hat{x}}_{k-1}^{+}$ is the previous time estimation value of the state. $P_{k}^{-}$ is the one-step prediction of the state covariance matrix, and $P_{k-1}^{+}$ is the previous time estimation of the state covariance matrix. $F_{k}^{-}$ is the Jacobian matrix of the state equation solved according to ${\hat{x}}_{k}^{-}$, and $Q_{k}$ is the state noise matrix.

(7) $$\left\{\begin{array}{c} {\hat{x}}_{k}^{-}=f\left({\hat{x}}_{k-1}^{+}\right)\\ P_{k}^{-}=F_{k}^{-}P_{k-1}^{+}F{{}_{k}^{-}}^{T}+Q_{k}^{}\\ F_{k}^{-}={\left.\frac{\partial f\left(x\right)}{\partial x}\right|}_{x={\hat{x}}_{k}^{-}} \end{array}\right.\;$$

After the time update shown in Figure 2, the measurement update in different angular motion states is performed separately. When the output of gyroscope in MEMS IMU-H or IMU-L is almost zero or a small value, that means there is no angular motion, the measurement update process is as shown in Equation (8), where I is a unit matrix.

(8) $$\left\{\begin{array}{c} \kappa_{k}=P_{k}^{-}H_{}^{T}{\left(H_{}^{}P_{k}^{-}H_{}^{T}+R_{k}\right)}^{-1}\\ {\hat{x}}_{k}^{+}={\hat{x}}_{k}^{-}+\kappa_{k}\left(z_{k}^{}-H{\hat{x}}_{k}^{-}\right)\\ P_{k}^{-}=\left(I-\kappa_{k}H\right)P_{k}^{-}{\left(I-\kappa_{k}H\right)}^{T}+\kappa_{k}R_{k}{\kappa_{k}}_{}^{T} \end{array}\;\right.\;$$

When there is no angular motion, scale factor errors and misalignment errors are not observable, thus current time estimates of them remain as the state one-step prediction values. The state estimates and covariance matrix estimates for scale factor errors and misalignment errors are shown in Equation (9).

(9) $$\left\{\begin{array}{c} {\hat{x}}_{k}^{+}\left(7:12,1\right)={\hat{x}}_{k-1}^{+}\left(7:12,1\right)\\ P_{k}^{+}\left(7:12,7:12\right)=P_{k-1}^{+}\left(7:12,7:12\right) \end{array}\right.$$

As mentioned above, in the multi-rotor UAV, there is no special large maneuver, no more than the measurement range of the gyroscope in MEMS IMU-H, and when the gyroscope in MEMS IMU-L is also fault-free, the angular rate fusion can be performed according to the above fusion process. However, in practical applications, the two types of gyroscopes will inevitably have abnormal output, so Section 2.3 will propose corresponding detection and isolation methods for the abnormal output that occurs during the fusion process.

### 2.3. Performance Enhancement Method of Angular Rate Measurement Based on Measurement Noise Adaptive Adjustment

It can be seen from the redundant MEMS IMU angular rate fusion process that abnormal angular rate measurements of MEMS IMU-H or IMU-L would affect the accuracy and stability of the fused angular rate. To this end, a performance enhancement method of angular rate measurement based on abnormal information diagnosis and measurement noise adaptive adjustment is proposed in this section.

The angular rate output in MEMS IMU-H is more stable than that in IMU-L, so it can be assumed that it has a low probability of failure within the measurement range. Since the gyroscope in MEMS IMU-L has a wider measurement range than that in the IMU-H, it would not have an over-range anomaly. Therefore, there are two kinds of abnormal conditions in the above filter: (1) the gyroscope in MEMS IMU-L is out of order; (2) the gyroscope in MEMS IMU-H over-range is out of range.

Both abnormal conditions lead to the abnormal modulus of the measurement innovation $\delta_{k}^{}\;=\;z_{k}^{}-H{\hat{x}}_{k}^{-}$ in Equation (8). In addition, then the abnormal angular rate input is detected by designing the detection threshold of the innovation modulus. Based on judging the measurement anomaly, it is also necessary to determine whether the gyroscope in MEMS IMU-H is out of range, to accurately locate the cause of the abnormality. Thus, the design anomaly detection criterion is as shown in Equation (10), where σ is the detection threshold of the innovation modulus and $\omega_{\max}^{}$ is the maximum range of the gyroscope in MEMS IMU-H.

(10) $$\begin{array}{c} if\left|\delta_{k}^{}\right|\geq \sigma \;\mathrm{and}\;\left|\omega_{axis\_high}^{}\right|\leq 0.9\omega_{\max}^{}\;,\;\omega_{axis\_low}^{}\;\mathrm{is}\;\mathrm{fault}\;,\;axis\;=\;x,y,z\\ if\left|\delta_{k}^{}\right|\geq \sigma \;\mathrm{and}\;\left|\omega_{axis\_high}^{}\right|>0.9\omega_{\max}^{}\;,\;\omega_{axis\_high}^{}\;\mathrm{is}\;\mathrm{fault}\;,\;axis\;=\;x,y,z \end{array}$$

Due to the influence of the range nonlinearity during the MEMS gyroscope processed, the measurement error of the MEMS gyroscope would increase significantly when it was close to 90% of the maximum measurement range. Therefore, 90% of the maximum range of the MEMS Gyro-H is selected in Equation (10) as the over-range judgment condition.

When $\omega_{axis\_low}$ is fault in Equation (10), the fusing angular rate is the output of gyroscope in MEMS IMU-H. In addition, when $\omega_{axis\_high}$ is fault, the measurement noise in the corresponding axis is adjusted to a preset maximum value to weaken the influence of the gyroscope in MEMS IMU-H on the fusion result.

If it is judged that there is no abnormal information in the fusion, it is still necessary to optimize the redundant angular rate fusion method. The reason is that when $k_{\omega }$ is in $70\%<k_{\omega }\leq 90\%$, which is the ratio of $\omega_{axis\_high}$ and $\omega_{m}ax$, the gyroscope measurement of MEMS IMU-H becomes less reliable. In order to ensure the stability of the fusion angular rate, the measurement noise needs to be adaptively increased according to $k_{\omega }$, thereby gradually weakening the influence of the gyro-H instability on the fusion result. If $k_{\omega }\leq 70\%$, the set minimum measurement noise would be used to increase the correction of MEMS Gyro-L errors by gyro-H and ensure the fast convergence of gyro-L error parameters. The adaptive adjustment process of the measurement noise can be expressed as Equation (11), where *V* represents mean square error of the measurement noise, $V_{min}$ and $V_{max}$ are the preset minimum and maximum value.

(11) $$\begin{array}{c} \left|V_{axis}\right|=V_{\min},k_{\omega }^{}\leq 70\%\\ \left|V_{axis}\right|=5\left(k_{\omega }^{}-0.7\right)\left(V_{\max}-V_{\min}\right)+V_{\min},70\%<k_{\omega }^{}\leq 90\%\\ \left|V_{axis}\right|=V_{\max},k_{\omega }^{}>90\% \end{array}$$

**Figure 3:**
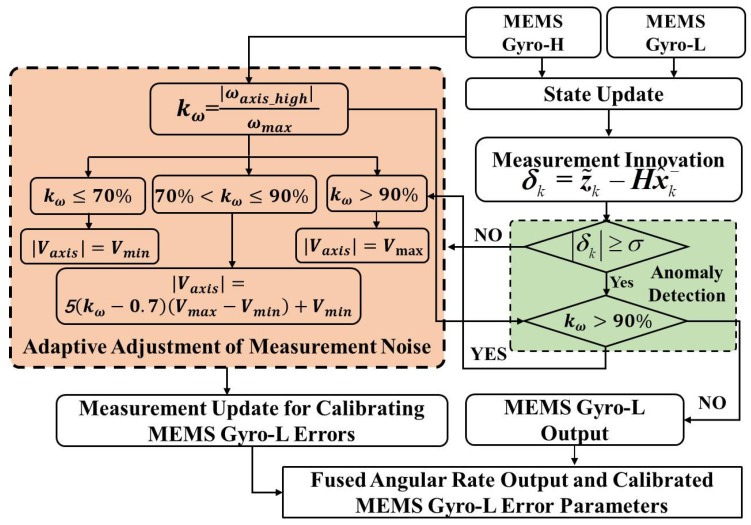
Optimal angular rate fusion process based on the diagnosis of abnormal information and adaptive adjustment of measurement noise.

The optimal angular rate fusion process based on the diagnosis of abnormal information and adaptive adjustment of measurement noise is shown in Figure 3. The angular rate fusion method proposed in Section 2 can not only improve the accuracy and stability of angular rate measurement, but also extend the angular rate measurement range. The proposed method will be validated in Section 3.

## 3. Validation and Analysis

To verify the proposed performance enhancement method of angular rate measurement based on redundancy MEMS IMUs, the experiments were designed from the following two aspects. (1) Digital simulation experiment is designed, in which the outputs of gyro in MEMS IMU-H and IMU-L are simulated in different dynamic situations. (2) The proposed method is verified in dynamic experiments based on the designed redundant MEMS IMUs.

### 3.1. Verification Experiment Based on Simulation Data

In the simulation verification, the gyroscope outputs of MEMS IMU-L and IMU-H are simulated according to their error models. As the gyroscope of MEMS IMU-H has high stability and high accuracy, especially after calibration, the scale factor error and misalignment error are almost zero, it is simulated using a model with ideal output plus white noise [21]. The gyroscope of MEMS IMU-L simulation model includes bias, scale factor error, misalignment error and white noise, and the first three errors are added in the form of random constants. In addition, the simulation experiment also needs to add the abnormal gyro-L and gyro-H output, to verify more fully the proposed method.

#### 3.1.1. Simulation Condition

The error parameters and measurement range of gyroscope in MEMS IMU-H and IMU-L are set as shown in Table 1.

**Figure 4:**
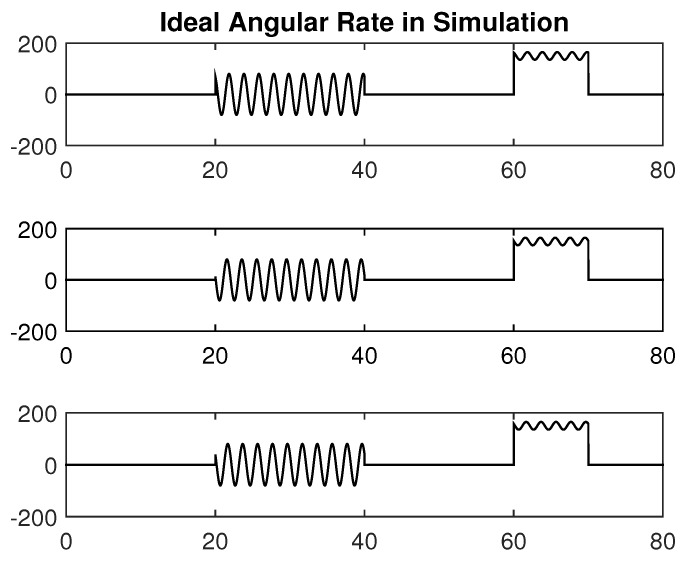
Ideal triaxial angular rate.

**Figure 5:**
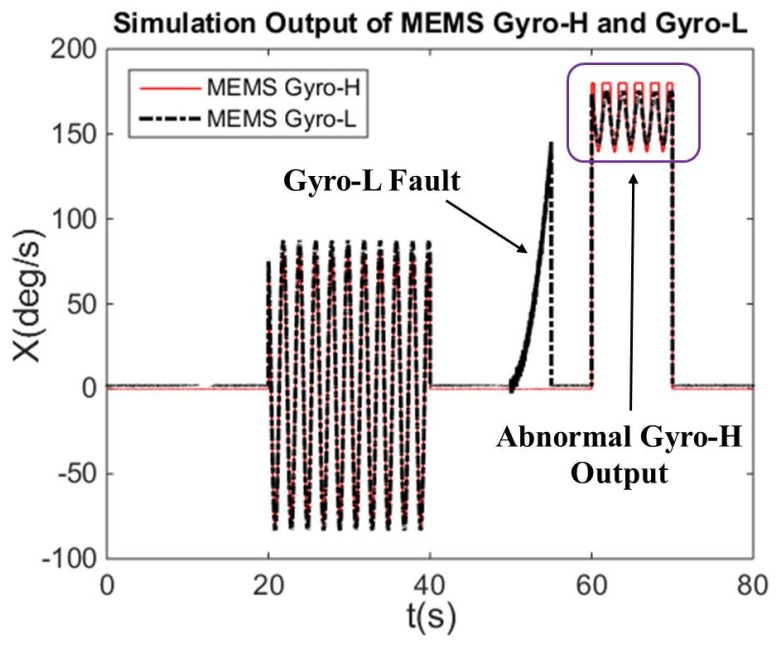
*X*-axis gyroscope output in MEMS IMU-H and IMU-L.

The curves of the ideal triaxial angular rate change with time are shown in Figure 4. The corresponding curves of X-axis gyroscope output in MEMS IMU-H and IMU-L are shown in Figure 5. In addition, the output of the ideal triaxial angular rate for each time duration is set in Table 2.

In Figure 5, during 0–40 s, the gyroscope error parameters in MEMS IMU-L are calibrated online. The 40s–60s verifies the effectiveness of the performance enhancement method based on redundancy configuration when the gyroscope of MEMS IMU-L output is abnormal. Then the 60–70 s verifies the effectiveness of the angular rate fusion method by adaptively adjusting the measurement noise when the gyroscope of MEMS IMU-H exceeds 70% of the range.

#### 3.1.2. Simulation Verification Results

**Figure 6:**
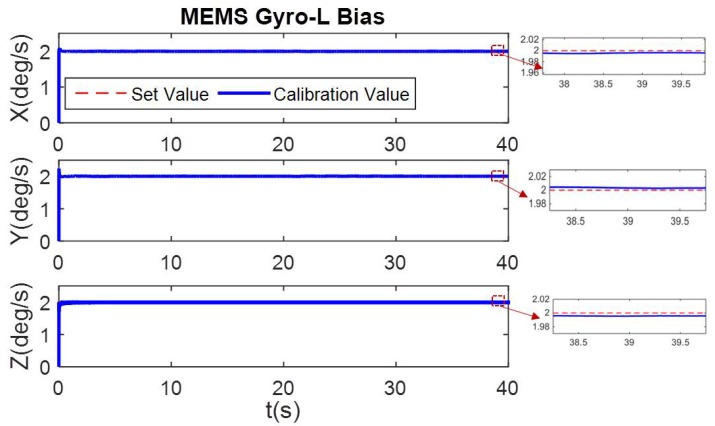
Online calibration result of MEMS Gyro-L bias.

**Figure 7:**
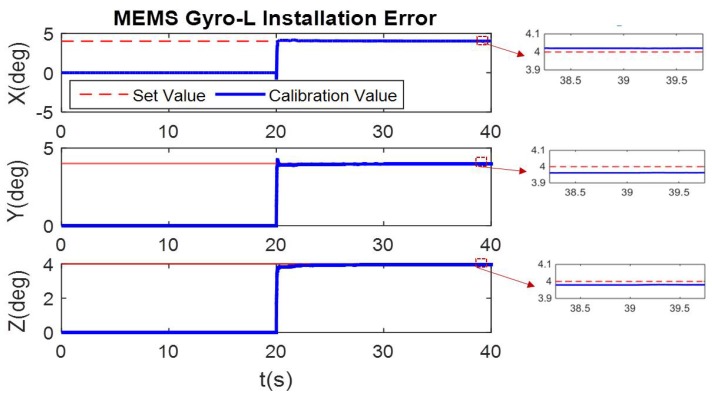
Online calibration result of MEMS Gyro-L misalignment error.

**Figure 8:**
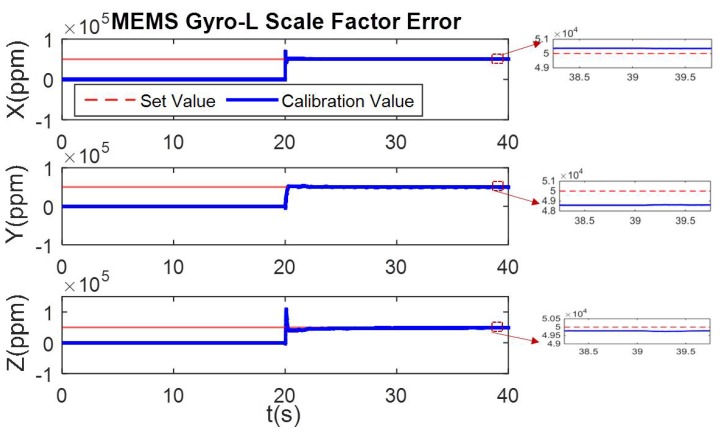
Online calibration result of MEMS Gyro-L scale factor error.

In the simulation condition, the first 20 s has non-angular motion, i.e., the tri-axis angular rate is zero. The calibration process of Figure 2 shows that the tri-axis gyroscope bias is calibrated, while scale errors and misalignment errors remain the initial setting value. Then in the 20–40 s, when the three axes have angular rate, all the error parameters can be calibrated. The calibration results are shown in Figure 6, Figure 7 and Figure 8.

The set value, the calibration result, and the calibration residual of each error parameter is shown in Table 3. It can be seen from Table 3 that the MEMS Gyro-L bias estimation accuracy is about 99.6%, the misalignment error estimation accuracy is about 97.3%, and the scale factor error estimation accuracy is about 96.5%. The calibration results show that in the angular rate fusion, the online calibration and compensation of these error parameters can effectively improve the angular rate measurement accuracy of MEMS IMU-L. In the actual system, the calibration and compensation effects of these errors would be affected by the bias stability of the redundant MEMS gyroscopes and the time synchronization of the acquired data. Thus, the accuracy of actual calibration results would be slightly worse than that of simulation calibration ones, but the error parameters of MEMS Gyro-L could be still compensated.

**Figure 9:**
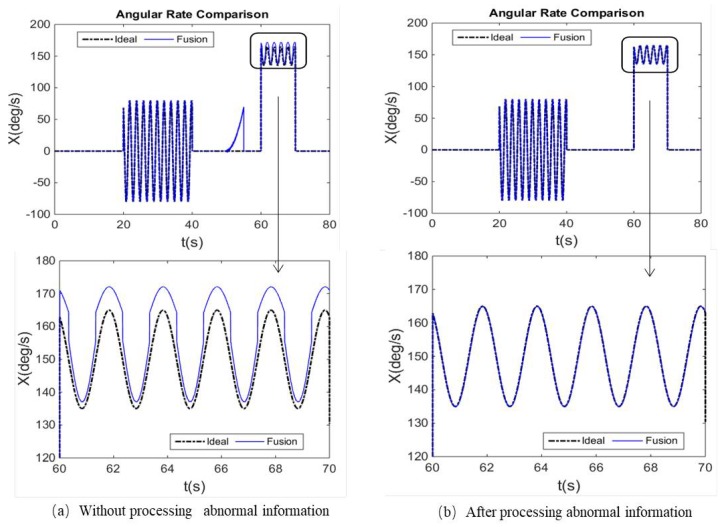
Comparison of the ideal and fusion angular rate. (**a**) Comparison of the ideal and fusion angular rate without processing abnormal information and (**b**) comparison of the ideal and fusion angular rate after processing abnormal.

**Figure 10:**
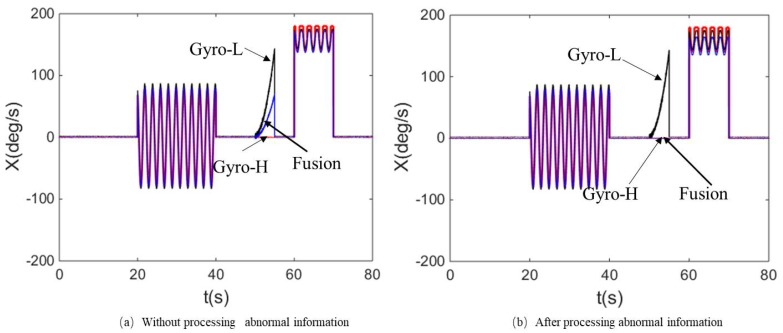
Comparison of the fusing angular rate and the output of MEMS Gyro-H and Gyro-L. (**a**) Comparison of the ideal and fusion angular rate without processing abnormal information and (**b**) comparison of the ideal and fusion angular rate after processing abnormal.

**Figure 11:**
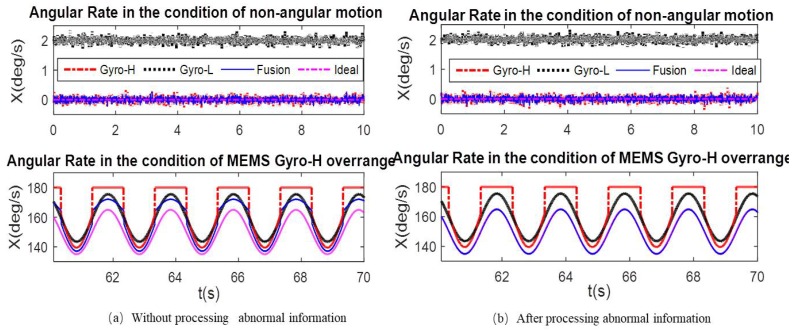
*X*-axis angular rate comparison in the condition of non-angular motion and MEMS Gyro-H over-range. (**a**) *X*-axis angular rate comparison in the condition of non-angular motion and MEMS Gyro-H over-range without processing abnormal information and (**b**) *X*-axis angular rate comparison in the condition of non-angular motion and MEMS Gyro-H over-range after processing abnormal information.

To more fully verify the influence of the abnormal information processing and the measurement noise adaptive adjustment on the angular rate fusion performance, Figure 9a, Figure 10a and Figure 11a show that the comparison curves of the fused angular rate without processing abnormal information and adjusting measurement noise. Figure 9b, Figure 10b and Figure 11b show that the comparison curves of the fusing angular rate by processing abnormal information and adjusting measurement noise adaptively.

It can be seen from Figure 9a and Figure 10a that when the MEMS Gyro-L fails at 50~55 s, the fault information leads to erroneous fusion output. However, when the abnormal information is detected and isolated, the fusion result is stable as shown in Figure 9b and Figure 10b.

As seen from the partial enlarged views of Figure 9a and Figure 11a, when the ratio of the MEMS Gyro-H output and the range is greater than 70%, the output gradually becomes abnormal. Thus, if the measurement noise was not adaptively adjusted, the accuracy and reliability of the fused output would be affected, thereby deviating from the ideal angular rate. When the measurement noise adaptive adjustment loop is added, the accuracy and reliability of the fusion angular rate can be effectively improved, as shown in Figure 9b and Figure 11b. Meantime, it can be seen from the partial enlarged view of Figure 11a,b that the fusion angular rate stability and accuracy are higher than those of the MEMS Gyro-H and Gyro-L without the angular motion.

Seen from the analysis of he above simulation results, we can conclude that the accuracy and stability of the angular rate measurement after the fusion are improved, and the measurement range is far beyond the range of the MEMS Gyro-H. The design of the simulation experiment fully validates that the heterogeneous MEMS IMU data fusion can effectively improve the angular rate measurement accuracy and stability and broaden the angular measurement range, by processing the abnormal angular rate and adjusting adaptively the measurement noise.

### 3.2. Verification Experiment Based on Sensor Measurement Data

To further demonstrate the proposed performance enhancement method of angular rate measurement, a redundant MEMS IMU module equipped with the industrial-grade and consumer-grade MEMS IMU has been designed. The measured outputs of the two types of gyroscopes under different dynamic conditions are collected to test the proposed method.

#### 3.2.1. Experiment Setup Based on the Designed Redundancy MEMS IMU

**Figure 12:**
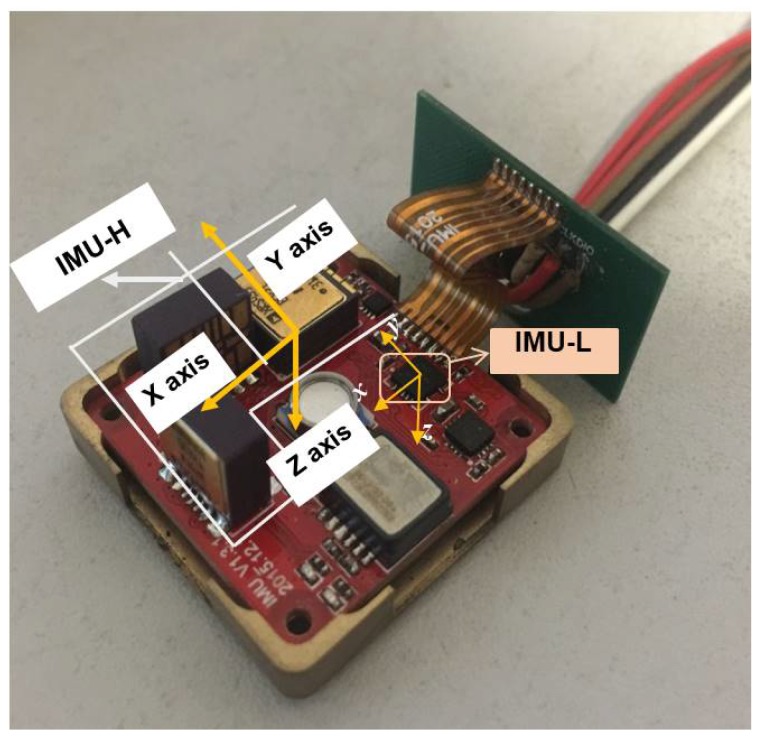
A redundant MEMS IMU module.

A redundant MEMS IMU module as shown in Figure 12 is designed to perform the verification based on sensor measurement data.

In Figure 12, MEMS IMU-H contains three single-axis high-precision gyroscopes, while the MEMS IMU-L has a three-axis low-precision gyroscope. It can be seen from the photo that the two types of MEMS IMU are installed in parallel. The measurement range of MEMS Gyro-H is 150 deg/s and the bias stability is 16.0 deg/h.

#### 3.2.2. Experiment Results Analysis

**Figure 13:**
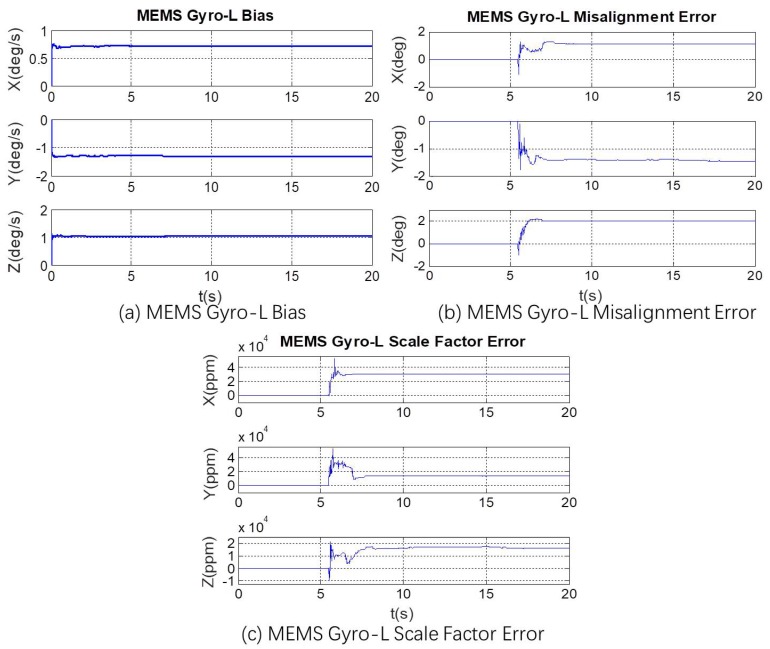
Online calibration results of MEMS Gyro-L error parameters. (**a**) Online calibration results of MEMS Gyro-L bias and (**b**) online calibration results of MEMS Gyro-L misalignment error and (**c**) online calibration results of MEMS Gyro-L scale factor error.

Based on the static and dynamic MEMS Gyro-H and Gyro-L outputs acquired from the redundant MEMS IMU module, the calibration results of MEMS Gyro-L error parameters are shown in Figure 13. It can be seen from the figures that bias, misalignment errors and scale factor errors are all effectively calibrated.

**Figure 14:**
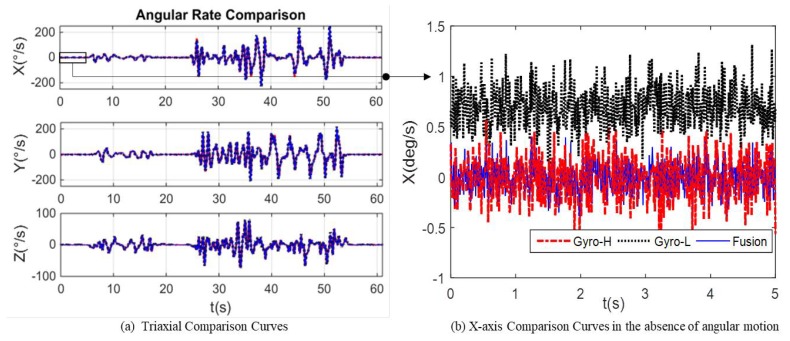
(**a**) Comparison of fusion results and output of the redundant MEMS IMU module and (**b**) *X*-axis comparison curves in the absence of angular motion.

The MEMS Gyro-L and Gyro-H outputs of the redundant MEMS IMU module are fused by the proposed method, and the compared curve of the fusion result and output of the two types of MEMS gyroscopes is as shown in Figure 14.

It can be seen from Figure 13 that the MEMS Gyro-L error parameters are calibrated online within 0 20 s. In the absence of angular motion, the fused gyroscope output is shown in Figure 14b. In Figure 14b, the angular rate accuracy and stability after fusion are improved, which are consistent to the results of the simulation experiments.

**Figure 15:**
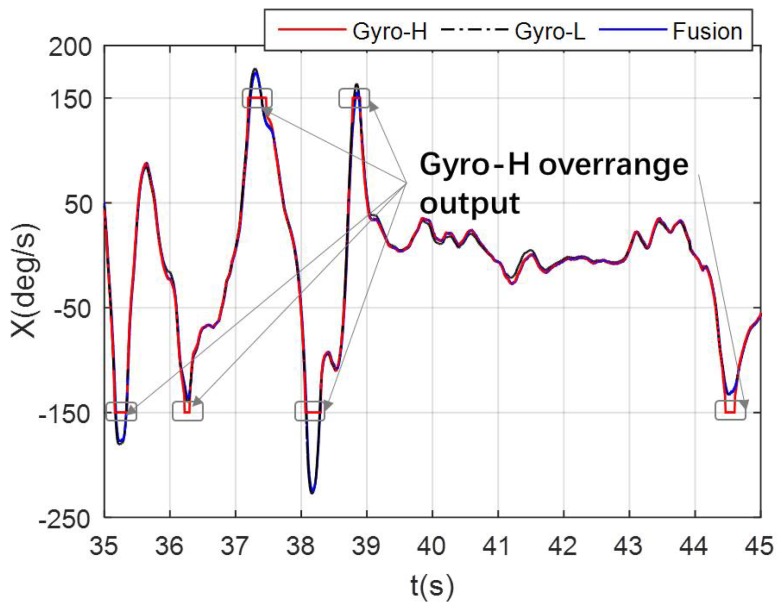
Comparison of fusion results and output of the redundant MEMS IMU module after adaptively adjusting measurement noise.

During the 25–55 s of Figure 14a, the gyroscope output of the MEMS IMU-H reaches more than 70% of the range. Thus, it is necessary to adaptively adjust the measurement noise to ensure the accuracy and stability of the fusion angular rate. The partial contrast results of the fusion angular rate and the gyroscope output of the MEMS IMU-H and IMU-L after adaptively adjusting measurement noise are shown in Figure 15. It can be seen from the partial enlarged view of Figure 15 that even if the MEMS IMU-H cannot output the correct angular rate due to over-range, after the adaptive adjustment of the measurement noise, the accuracy and reliability of the fused angular rate output can be ensured.

It can be seen from the above analysis that the angular rate accuracy and stability after fusion are improved, and the angular rate measurement range is extended, compared to only using the industrial-grade narrow-range MEMS IMU, which is consistent to conclusions of the simulation experiment. Therefore, whether it is the digital simulation or actual data verification experiment, the effectiveness of the proposed angular rate measurement performance enhancement method is fully demonstrated.

## 4. Conclusions

According to the low-cost, wide-range, and accurate measurement requirement for MEMS IMU on the multi-rotor UAV, this paper designs a heterogeneous parallel redundancy configuration, which combines an industrial-grade MEMS IMU(IMU-H) with consumer-grade MEMS IMUs. In the redundant configuration, the angular rate fusion model is established based on online calibrating gyro errors of MEMS IMU-L, and the fusion algorithm to deal with MEMS gyroscope abnormal outputs is proposed. By the designed experiment based on the simulation data and the sensor measurement data, the proposed method is proved that it can effectively improve the angular rate measurement accuracy and reliability of MEMS IMU. The proposed method can not only improve the multi-rotor UAV control reliability and anti-jamming capability, but also can be applied on robotics and self-driving vehicles.

## Figures and Tables

**Table 1:** Simulation parameter settings of the gyroscope in industrial-grade and consumer-grade Microelectromechanical System Inertial Measurement Unit (MEMS IMU-H and IMU-L).

MEMS Gyro	White Noise	Bias	Scale Factor Errors	Installation Errors	Measurement Range
gyro-H	0.1 deg/s	-	-	-	180 deg/s
gyro-L	0.1 deg/s	2 deg/s	data	20 deg/s	data

**Table 2:** Ideal triaxial angular rate of each time duration.

*t*	$\omega_{x}$ (deg/s)	$\omega_{y}$ (deg/s)	$\omega_{z}$ (deg/s)
0–20 s	0	0	0
20–40 s	80 cos (πt + 30)	2	data
40–60 s	0	0	0
60–70 s	150 + 15 cos (πt + 30)	150 + 15 cos (πt + 80)	150 + 15 cos (πt + 60)
70–80 s	0	0	0

**Table 3:** Calibration result of gyroscope parameters in MEMS IMU-L.

Error Parameter	Set Value	Calibration Result	Residual
$\varepsilon_{x}$	2 deg/s	1.994 deg/s	$6.2\times 10^{-3}$ deg/s
$\varepsilon_{y}$	2 deg/s	1.999 deg/s	$1.1\times 10^{-3}$ deg/s
$\varepsilon_{z}$	2 deg/s	2.002 deg/s	$2.3\times 10^{-3}$ deg/s
$\theta_{gx}$	4 deg	4.027 deg	$2.7\times 10^{-2}$ deg
$\theta_{gy}$	4 deg	3.990 deg	$9.8\times 10^{-3}$ deg
$\theta_{gz}$	4 deg	3.975 deg	$2.5\times 10^{-2}$ deg
$K_{gx}$	$5\times 10^{4}$ ppm	$5.035\times 10^{4}$ ppm	$3.5\times 10^{2}$ ppm
$K_{gy}$	$5\times 10^{4}$ ppm	$4.974\times 10^{4}$ ppm	$2.6\times 10^{2}$ ppm
$K_{gz}$	$5\times 10^{4}$ ppm	$4.868\times 10^{4}$ ppm	$1.3\times 10^{3}$ ppm
